# Melatonin treatment increases the transcription of cell proliferation-related genes prior to inducing cell death in C6 glioma cells *in vitro*

**DOI:** 10.3892/ol.2013.1413

**Published:** 2013-06-18

**Authors:** JIAGUI QU, JOSHUA D. RIZAK, XIAOMIAO LI, JIEJING LI, YUANYE MA

**Affiliations:** 1 School of Life Sciences, University of Science and Technology of China, Hefei, Anhui 230026;; 2 State Key Laboratories of Brain and Cognitive Science, Kunming, Yunnan 650223;; 3 Genetic Resources and Evolution, Kunming Institute of Zoology, Chinese Academy of Sciences, Kunming, Yunnan 650223;; 4 State Key Laboratory of Brain and Cognitive Science, Institute of Biophysics, Chinese Academy of Sciences, Beijing 100101, P.R. China

**Keywords:** melatonin, glioma, Nestin, Bmi-1, Sox2, cell viability, survival

## Abstract

A number of studies have suggested that melatonin possesses anticancer properties. However, conflicting data exists with regard to the role of melatonin in the treatment of cancer. In the present study, the effects of melatonin on the transcriptional regulation of three genes associated with cell proliferation (Nestin, Bmi-1 and Sox2), and on C6 glioma cell survival and viability, were investigated *in vitro* to evaluate the use of melatonin in cancer therapy. Melatonin was shown to increase the mRNA levels of Nestin, Bmi-1 and Sox2 in a similar pattern, with the highest mRNA levels noted at a concentration of 3 mM. At higher concentrations of melatonin (5 mM), the mRNA levels of Nestin, Bmi-1 and Sox2 were reduced from their peak levels, and were correlated with changes observed in immunofluorescence morphology studies, cell viability and survival assays. Immunofluorescence studies of Nestin-stained cells demonstrated that treatment with a higher concentration of melatonin (3 and 5 mM) led to the Nestin filaments condensing and rearranging around the cell nuclei, and an alteration in the cell morphology. C6 cell viability was also significantly decreased at 3 mM melatonin, and cell death was observed at 5 and 10 mM melatonin. These results suggested that Nestin, Bmi-1 and Sox2 were strongly correlated with the survival of C6 cells following treatment with melatonin, and that high therapeutic concentrations of melatonin (>5 mM) were required to induce cell death. These findings suggested that the implementation of melatonin in the treatment of glioma and other types of cancer may be inhibited by conflicting cell growth signals in cells. Therefore, adjunct therapy is required to improve the efficacy of melatonin in the treatment of cancer.

## Introduction

Melatonin, a neurohormone secreted predominately by the pineal gland in animals, has been suggested to possess anticancer properties in a number of studies (1). Physiologically, melatonin is secreted at low nanomolar concentrations and has been demonstrated to be an effective antioxidant, free radical scavenger (2) and regulator of antioxidant genes (3,4). This has led to the long-term or daily administration of melatonin as a pharmacological supplement at concentrations almost one million-fold higher than the physiological levels (5). The supplementation of melatonin has been shown to alleviate the symptoms of several degenerative diseases associated with aging [including Alzheimer’s and Parkinson’s disease (6,7)], to reduce the incidence of malignant tumors *in vivo* (8) and to increase the survival time of patients with glioblastomas treated with radiotherapy (9). Melatonin has also been demonstrated to suppress the growth, migration and invasion of C6 glioma cells *in vitro* by modulating numerous oxidative stress pathways (10–12), and to exert an apoptotic effect in several types of cancer (1). These findings, in addition to the fact that no significant side effects have been identified with the use of melatonin (13), have formed the basis of the hypothesis that melatonin may have a beneficial efficacy in the prevention of cancer (5), and may be used to further supplement adjunct therapies in the prevention or treatment of numerous types of cancer, including glioma.

However, melatonin has also been demonstrated to inhibit apoptotic pathways in a number of cell types (14–16), as well as having a role in stem cell proliferation and the epigenetic regulation of neural cell growth *in vitro* (17). These findings suggest that the modulation of melatonin may not be limited to treating cancer or exerting an apoptotic effect, and that numerous intracellular mechanisms may be involved in promoting cancer cell survival.

Melatonin has been shown to induce the expression of Nestin, a type VI intermediate filament protein, in the C17.2 neural stem cell line (17). Nestin is a marker of neural stem cells. It is transiently expressed during the development of the nervous system and is important in the proliferation and the non-differentiated status of neural stem cells (18,19). Previous studies have demonstrated the involvement of Nestin in the development of cancer, and have suggested that several types of cancer that present with Nestin-positive tumors have a poor prognosis (20–24). The general differentiation status of tumor cells has been shown to be an important factor directly correlated with the malignancy of tumors (25,26). The finding that melatonin affects the expression of Nestin suggests that melatonin may have an effect on stem cell differentiation which promotes the development of cancer.

In the present study, the effect of pharmacological concentrations of melatonin on the proliferation, growth and survival of C6 glioma cells was evaluated *in vitro* to examine the role of melatonin in the treatment of cancer. C6 glioma cells provided a good model to investigate the effect of melatonin on cell growth *in vitro*, as these cells express two types of extracellular melatonin receptors (MT1 and MT2) and are susceptible to modulation by melatonin at pharmacological concentrations (10,11). The transcription of Nestin, along with the transcription of two other genes that are important to nervous system development (Bmi-1 and Sox2) (27,28), were used as markers of cell proliferation to evaluate the role of melatonin on glioma cell differentiation and proliferation. Bmi-1 and Sox2 were selected as cell proliferation markers in this study, as they possess similar roles in cell growth (27,29–31), have been implicated in cancer development (32–37) and have not been demonstrated to be affected by melatonin (38). The effect of melatonin on the cell morphology, viability and death of C6 glioma cells was additionally evaluated in comparison with the levels of transcription of these proliferation markers.

## Materials and methods

### Cell culture and treatment

C6 glioblastoma cells (C6 cells) were provided by the Cell Bank of Type Culture Collection of Chinese Academy of Sciences (Shanghai, China). C6 cells were cultured in F12K media (Sigma, St. Louis, MO, USA) supplemented with 15% equine serum (Hyclone, Beijing, China) and 2.5% fetal bovine serum (Hyclone). The cells were incubated at 37.5°C and 100% humidity in 95% air and 5% CO_2_.

All the experiments were conducted with cells at 70–80% confluence. C6 cells were cultured for 24 h prior to the addition of experimental treatments. The cells were treated with melatonin (Sigma) at concentrations of 0, 1, 3, 5 and 10 mM, and incubated for an additional 24 h. An equal volume of phosphate-buffered saline (PBS) was used as the vehicle treatment.

### Quantitative polymerase chain reaction (qPCR)

The mRNA transcription levels of the target genes (Nestin, Bmi-1 and Sox2) were analyzed with qPCR. Total RNA was extracted from C6 cells using the RNA Simple Total RNA kit (Tiangen, Beijing, China), according to the manufacturer’s instructions. The RNA was reverse-transcribed using the M-MLV First Strand kit (Invitrogen Life Technologies, Beijing, China). qPCR was performed using the iTaq™ Universal SYBR Green supermix (Bio-Rad, Hercules, CA, USA). The primers used for qPCR were as follows: Forward: 5′-ATGGGGTTCCTGTACTATCTG-3′ and reverse: 5′-GGTGTTGGCTCTCCTCTTTA-3′ for Nestin; forward: 5′-CCAAAGGAGGAGGTGAATGA-3′ and reverse: 5′-AGGTGTAAATGTAGGCAATGTC-3′ for Bmi-1; forward: 5′-TAGGGCTGGGAGAAAGAAGAG-3′ and reverse: 5′-ATCTGGCGGAGAATAGTTGG-3′ for Sox2; forward: 5′-GGGACCTGACAGACTACCTCA-3′ and reverse: 5′-ATTGCCGATAGTGATGACCTGA-3′ for β-actin. β-actin mRNA was used as a loading control.

### Immunostaining of cells

The C6 cells were trypsinized and mounted on glass coverslips precoated with polyornithine and laminin (Sigma). The cells were fixed with 4% paraformaldehyde and permeabilized with 0.4% Triton X-100 at room temperature (RT). Bovine serum albumin (5%; Amresco, Solon, OH, USA) was used to block the cells. The cells were then incubated with primary rabbit anti-Nestin polyclonal antibody (Millipore, Billerica, MA, USA) at 4°C overnight. The cells were incubated with PE-conjugated secondary antibody (anti-rabbit IgG-PE; Santa Cruz Biotechnology, Inc., Santa Cruz, CA, USA) at RT, and then stained with DAPI to identify all cell nuclei (39). Immunofluorescence and DAPI staining were detected by laser confocal scanning microscopy (IX81S1F-3, Olympus, Tokyo, Japan).

### MTT assay

The MTT assay (Amresco) was used to detect C6 cell viability (40). C6 cells cultured in 96-well plates were incubated with 10 *μ*l MTT (5 mg/ml) in a CO_2_ incubator for 4 h. The medium was then discarded using a suction pump and 100 *μ*l dimethylsulfoxide (DMSO) was added to each well to dissolve the MTT formazan crystals. The optical density at 570 nm was measured by an enzyme-linked immunosorbent detector (Bio-Rad, Kyoto, Japan).

### Flow cytometry analysis of cell survival status

The cell survival status was measured using the Annexin-V Apoptosis Detection kit (Becton-Dickinson, San Diego, CA, USA), according to the manufacturer’s instructions. Fluorescence was measured on a fluorescence activated cell sorter (the FACSVantageSE flow cytometer; Becton-Dickinson, Heidelberg, Germany) and analysis of the data was performed using WinMDI 2.9 software. Data are presented as dot plots of fluorescein isothiocyanate (FITC)-conjugated Annexin-V (X axis) and propidium iodide (PI; Y axis) staining.

### Statistical analysis

The results are presented as the mean ± standard deviation for at least three repeated individual experiments of each group. Analysis was performed with the SPSS 13.0 statistical software (IBM, New York City, NY, USA). Statistical differences were determined using one-way analysis of variance (ANOVA) for independent samples. P<0.05 was considered to indicate a statistically significant result.

## Results

### qPCR

**Figure 1.**
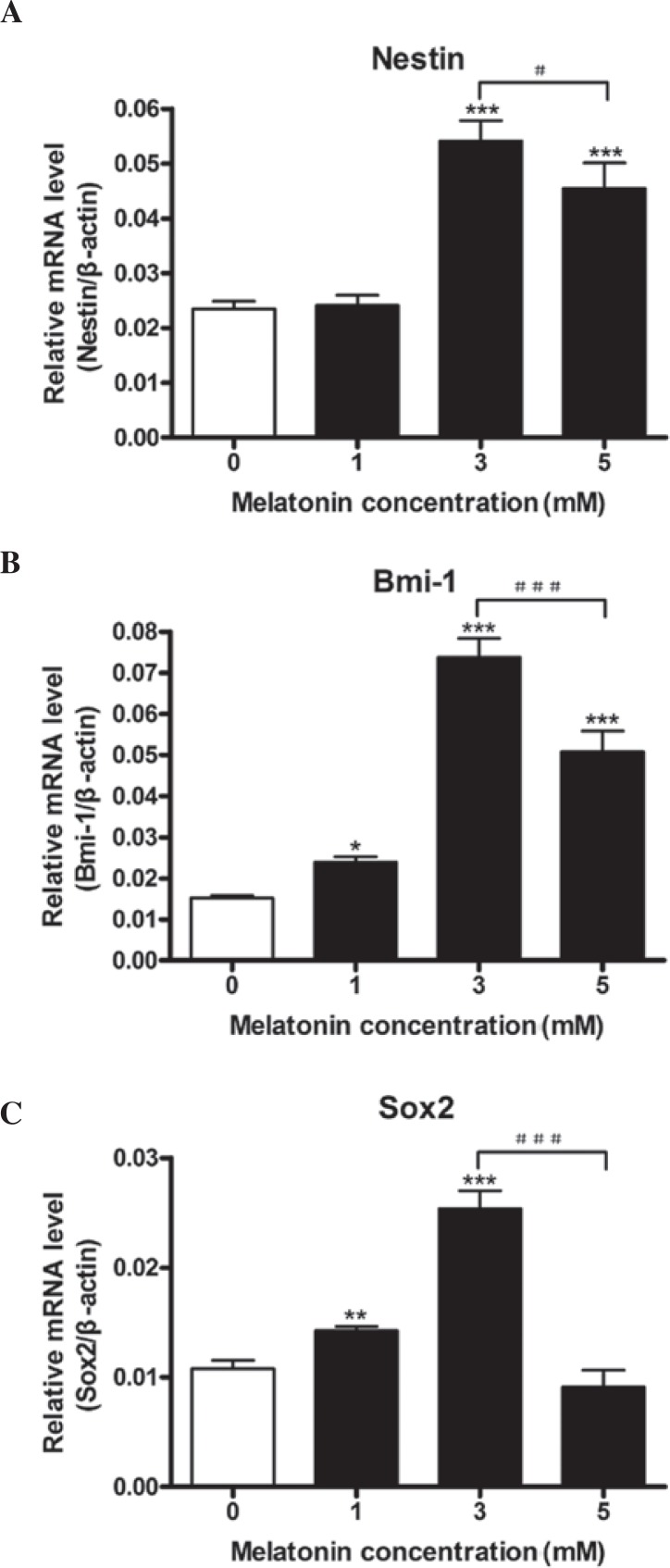
Melatonin modulates the mRNA levels of several cell differentiation markers in C6 glioma cells. C6 glioma cell cultures (n=3) were treated with different concentrations of melatonin (0, 1, 3 and 5 mM) for 24 h. (A) Nestin, (B) Bmi-1 and (C) Sox2 mRNA levels were quantified using quantitative polymerase chain reaction (qPCR). The results are normalized to the total β-actin mRNA levels and are presented as the mean ± SD. ^*^P<0.05, **P<0.01 and ^***^P<0.001 compared with the control. ^#^P<0.05 and ^###^P<0.001.

qPCR was used to study the transcription levels of Nestin, Bmi-1 and Sox2 in C6 glioma cells under various concentrations of melatonin. A general trend was observed for all three cell proliferation markers (Fig. 1A–C). Melatonin was demonstrated to increase the mRNA transcript levels in a dose-dependent manner at concentrations ≤3 mM; however, the increase in Nestin mRNA levels was not significant at the 1 mM dose. Bmi-1 demonstrated a five- to six-fold increase in mRNA transcript levels, whereas Nestin and Sox2 increased by approximately three-fold at their peak transcription levels (3 mM melatonin). At a concentration of 5 mM melatonin, all three mRNA transcript levels were decreased in comparison with their peak expression levels at 3 mM melatonin. At a concentration of 5 mM melatonin, Nestin and Bmi-1 levels were significantly reduced from their peak levels, yet remained increased from their basal levels. By contrast, Sox2 transcription returned to basal levels.

**Figure 2.**
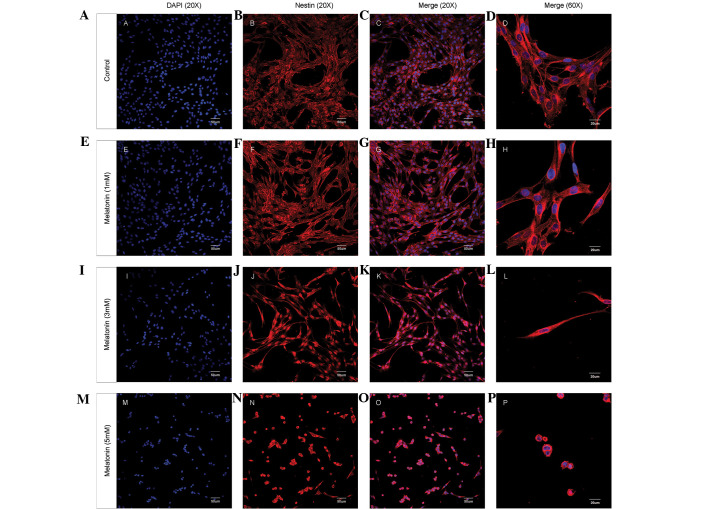
Melatonin treatment affects the protein distribution of Nestin and the morphology of C6 glioma cells. C6 glioma cell cultures were treated with different concentrations of melatonin (0, 1, 3 and 5 mM) for 24 h. Immunofluorescence images of C6 glioma cells and Nestin protein distribution are shown. (A, E, I and M) DAPI nuclear staining (magnification, ×20); (B, F, J and N) Nestin protein immunofluorescence (magnification, ×20); (C, G, K and O) overlay (magnification, ×20) and (D, H, L and P) overlay (magnification, ×60). PE-conjugated secondary antibody was used as the immunofluorescent dye.

The alterations in the mRNA transcript levels of these cell proliferation markers with respect to the concentration of melatonin were similar to the changes in the cell morphology of glioma cells and the distribution pattern of Nestin expression (Fig. 2). Untreated C6 cells exhibited a normal morphology with Nestin filaments dispersed in the cytoplasm and around the cell nuclei (Fig. 2A–D). Following treatment with 3 mM melatonin, the cells became longer and thinner, and the Nestin filaments began to condense around the cell nuclei (Fig. 2I–L). The majority of the cells retracted to a round morphology and the Nestin filaments became condensed around cell nuclei following treatment with 5 mM melatonin (Fig. 2M–P).

**Figure 3.**
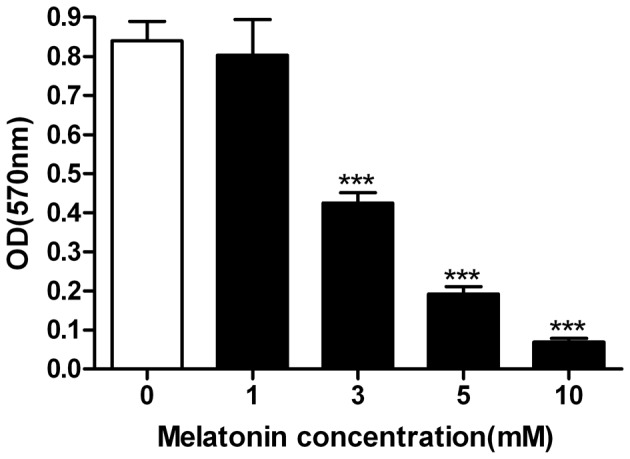
Melatonin decreases C6 glioma cell viability in a dose-dependent manner. C6 glioma cell cultures (n=10) were treated with different concentrations of melatonin (0, 1, 3, 5 and 10 mM) for 24 h. Cell viability was measured with the MTT assay. The optical density (OD) at 570 nm represents the cell viability. The results are presented as the mean ± SD. ^***^P<0.001 compared with the control.

**Figure 4.**
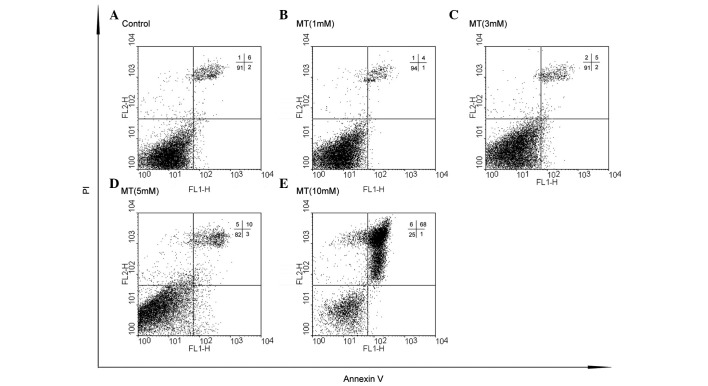
High therapeutic concentrations of melatonin induce cell death in C6 glioma cells. (A–E) C6 glioma cell cultures were treated with different concentrations of melatonin (0, 1, 3, 5 and 10 mM) for 24 h. The cells were marked by a fluorescein isothiocyanate (FITC)-conjugated Annexin-V and propidium iodide (PI) double stain, and were analyzed by flow cytometry. The FL1 channel was used to detect Annexin-V-FITC staining and the FL2 channel was used to detect PI staining.

The MTT assay and flow cytometry analysis were then performed to examine the effect of melatonin on C6 glioma cell viability and survival. At a concentration of 1 mM, melatonin exerted no effect on the viability of C6 cells (Fig. 3), which was consistent with the results of previous studies (10). However, 3 mM melatonin was demonstrated to reduce the viability of C6 cells to ∼50%, while 10 mM melatonin almost completely suppressed the cell viability (Fig. 3). Similarly, a small number of dead cells were observed when the glioma cells were treated with 0, 1 or 3 mM melatonin (Fig. 4A–C); however, these numbers increased with higher melatonin concentrations. At a concentration of 5 mM, melatonin was shown to induce low levels of cell death, while 10 mM melatonin was shown to induce a significant increase in cell death (Fig. 4D–E).

## Discussion

The present study investigated the effect of pharmacological concentrations of melatonin on C6 glioma cell survival and viability *in vitro,* and evaluated the role of melatonin on the transcriptional regulation of three genes (Nestin, Bmi-1 and Sox2) involved in the development of the nervous system and cancer progression. Melatonin was demonstrated to increase the mRNA levels of Nestin, Bmi-1 and Sox2 in a similar pattern, peaking at 3 mM melatonin. Immunofluorescence studies of Nestin-stained cells suggested that Nestin filaments condensed and rearranged around cell nuclei following melatonin treatment (at 3 and 5 mM). These results corresponded with the findings that melatonin also significantly decreased C6 cell viability at 3 mM, and induced cell death at 5 and 10 mM. Overall, these findings suggested that Nestin, Bmi-1 and Sox2 were strongly correlated with the survival of C6 cells following melatonin treatment, and that high therapeutic concentrations of melatonin (>5 mM) were required to induce cell death.

Melatonin possesses a wide variety of physiological functions, ranging from the regulation of the circadian rhythm (41) to acting as a potent antioxidant (2–4). A number of cellular membrane receptors are regulated by melatonin, including nuclear and extracellular membrane receptors (1), which may partially account for the differential responses to melatonin observed in normal and tumor cells. For example, the neural stem cell line C17.2 has been shown to increase the transcription rate of Nestin at physiological concentrations of melatonin (1 nM) (17), suggesting that melatonin has a role in stem cell proliferation in healthy tissues. The finding that therapeutic concentrations of melatonin (3 mM) were required to induce significant changes in the expression levels of Nestin in C6 glioma cells suggests differences between the cell lines; however, this result does not imply that glioma tissues are less responsive to melatonin. It has been shown that nanomolar concentrations of melatonin induced the transcription of glial cell line-derived neurotrophic factor (GDNF) mRNA in C6 glioma cells (42), and that micromolar concentrations of melatonin protected C6 glioma cells from amyloid-β-induced apoptosis (43). These findings support the hypothesis that the mechanism by which melatonin regulates cell growth is affected by the cell type and the concentration of melatonin. In glioma tumors, these functions may be involved in promoting cell growth, regardless of the fact that melatonin is proposed to be a potential apoptotic agent for cancer treatment (5).

Notably, Bmi-1 and Sox2 were upregulated at therapeutic concentrations of melatonin in the glioma cell line. Bmi-1 overexpression has been demonstrated to induce epithelial-mesenchymal transition, to promote tumor metastasis (32,33) and to cause radioresistance in cancer therapy (44). Sox2 has also been shown to be a marker of malignancy and is important in tumor cell progression (34–37). Notably, a previous study demonstrated that melatonin (100 *μ*M) had no effect on the transcription of Sox2 in a proliferating murine embryo stem cell line (38), which suggested that the induced transcription of Sox2 was the result of either a differential response to a higher concentration of melatonin or a specific response of the cancer-derived C6 glioma cell line. In either case, the increase in the rate of proliferation in the murine stem cell line and the increased levels of Sox2 expression indicated the potential of melatonin to affect cell growth at higher concentrations. These results imply that although differing cells have various response mechanisms to melatonin, the induced transcription of Nestin, Bmi-1 and Sox2 by melatonin is able to initiate a molecular proliferative response in C6 glioma cells that may contribute to the observed level of resistance to melatonin.

Although the proliferation of C6 cells was not observed, treatment with 3 mM melatonin was able to decrease cell viability and cause marginal disturbances to the cell morphology of glioma cells, despite increased transcript levels of Nestin, Bmi-1 and Sox2. This suggests that numerous mechanisms comprising distinct molecular pathways in glioma cells, which either promote cell growth or cell death, may be involved in the effects observed following melatonin treatment. The competition between the distinct pathways is highlighted by the correlation of increased levels of cell death, decreased cell viability and significant changes in cell morphology with decreased transcript levels of genes associated with cell proliferation at higher concentrations (5 mM) of melatonin, and the finding that 10 mM melatonin induced cell death.

Melatonin may possess anticancer properties for the treatment of several types of cancer, including glioma. The present study supports the requirement for additional or adjunct therapies in combination with melatonin treatment to fully inhibit the progression of cancer. The potential to target mechanisms that promote stem cell markers, including Nestin, Bmi-1 or Sox2, may provide adequate strategies to investigate which chemotherapies are most effective. Similar studies with regard to the activity of these genes in other neurological tumors may provide useful data on the cellular response to melatonin that promotes cell survival. Although the mechanisms that promote cell proliferation remain unclear and require further research, a number of studies have identified differential patterns of Nestin filament distribution and cell morphology in various types of neurogenic tumors (45–48), which may act as a template to assess the proliferation-promoting properties of melatonin compared with its potential apoptotic functions in various types of neurological cancer. Bmi-1 and Sox2 may also provide a similar template to assess the regulatory mechanisms and functions of melatonin in drug resistance. Future studies investigating these areas will aid in the screening of chemotherapies that function synergistically with melatonin, a non-toxic natural product with apoptotic properties, to induce cancer-specific cell death.
